# Childhood-related PTSD: the role of cognitions in EMDR and imagery Rescripting

**DOI:** 10.1080/20008066.2025.2460393

**Published:** 2025-02-10

Nele Assmann, Sophie A. Rameckersc, Anja Schaicha, Christopher W. Leed, Katrina Boterhoven de Haand, Marleen M. Rijkeboere, Arnoud Arntzc and Eva Fassbinder

*European Journal of Psychotraumatology*

Volume 15, Number 1, 2397890

https://doi.org/10.1080/20008066.2024.2397890

When it was first published online, Figure 1(a) contained an error. The correct Figure 1(a) is shown below:

**Figure 1.**
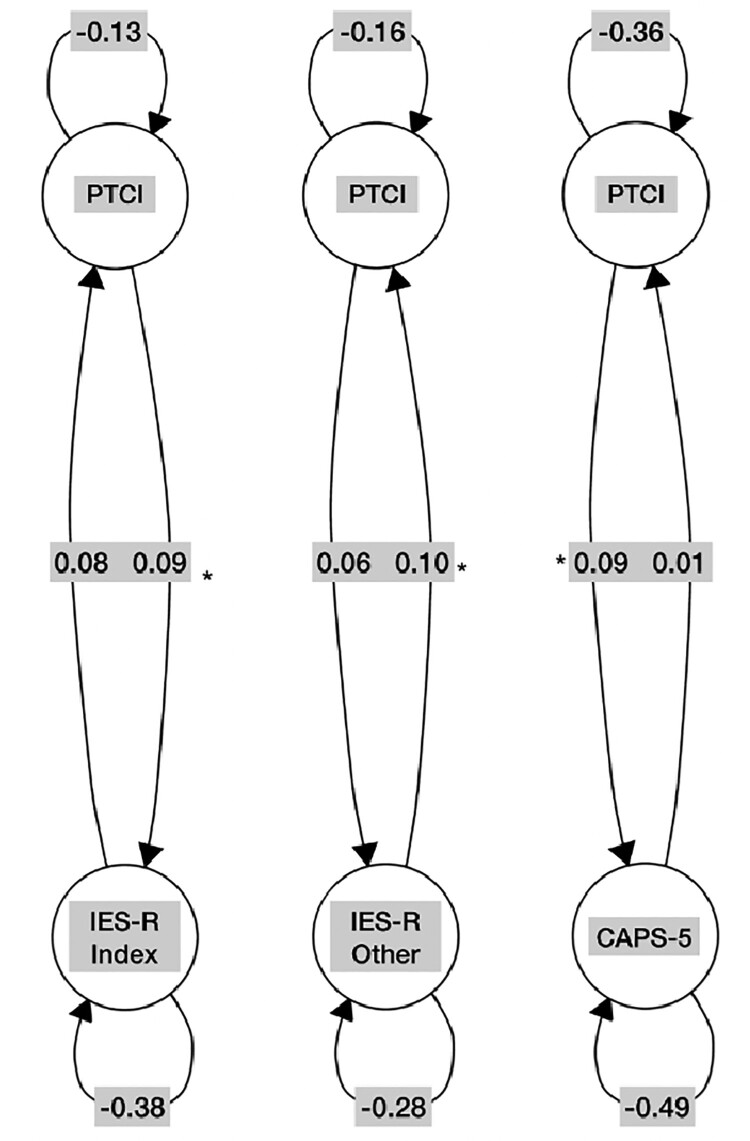
Graphic representation of the granger causality relationships for the PTCI subscales. Note: PTCI = Posttraumatic Cognitions Inventory, IES-R Index = Impact of Events Scale – Index trauma, IES-R Other = Impact of Events Scale – All other traumas, CAPS-5 = Clinical Administered PTSD Scale for DSM-5.

